# Disrupting Poly(ADP-ribosyl)ating Pathway Creates Premalignant Conditions in Mammalian Liver

**DOI:** 10.3390/ijms242417205

**Published:** 2023-12-06

**Authors:** Yaroslava Karpova, David J. Orlicky, Edward E. Schmidt, Alexei V. Tulin

**Affiliations:** 1 Department of Biomedical Sciences, School of Medicine and Health Sciences, University of North Dakota, 501 North Columbia Road, Grand Forks, ND 58202, USA; iaroslava.karpova@und.edu; 2 Koltzov Institute of Developmental Biology of Russian Academy of Sciences, 119334 Moscow, Russia; 3 Department of Pathology, University of Colorado School of Medicine, Aurora, CO 80045, USA; david.orlicky@cuanschutz.edu; 4 Microbiology & Cell Biology, Montana State University, Bozeman, MT 59718, USA; eschmidt@montana.edu; 5 Department of Microbiology & Immunology, Lewis Hall, Bozeman, MT 59718, USA; 6 Redox Biology Laboratory, University of Veterinary Medicine, 1078 Budapest, Hungary

**Keywords:** PARP1, PARG, poly(ADP-ribose) polymerase, poly(ADP-ribose) glycohydrolase, hepatocyte maturation, hepatocellular carcinoma, tumorigenesis, ductal response

## Abstract

Hepatocellular carcinoma (HCC) is a major global health concern, representing one of the leading causes of cancer-related deaths. Despite various treatment options, the prognosis for HCC patients remains poor, emphasizing the need for a deeper understanding of the factors contributing to HCC development. This study investigates the role of poly(ADP-ribosyl)ation in hepatocyte maturation and its impact on hepatobiliary carcinogenesis. A conditional *Parg* knockout mouse model was employed, utilizing Cre recombinase under the albumin promoter to target *Parg* depletion specifically in hepatocytes. The disruption of the poly(ADP-ribosyl)ating pathway in hepatocytes affects the early postnatal liver development. The inability of hepatocytes to finish the late maturation step that occurs early after birth causes intensive apoptosis and acute inflammation, resulting in hypertrophic liver tissue with enlarged hepatocytes. Regeneration nodes with proliferative hepatocytes eventually replace the liver tissue and successfully fulfill the liver function. However, early developmental changes predispose these types of liver to develop pathologies, including with a malignant nature, later in life. In a chemically induced liver cancer model, *Parg*-depleted livers displayed a higher tendency for hepatocellular carcinoma development. This study underscores the critical role of the poly(ADP-ribosyl)ating pathway in hepatocyte maturation and highlights its involvement in liver pathologies and hepatobiliary carcinogenesis. Understanding these processes may provide valuable insights into liver biology and liver-related diseases, including cancer.

## 1. Introduction

Hepatocellular carcinoma (HCC), the most common form of liver cancer, ranks worldwide as the third leading cause of cancer-related deaths overall and second for men [1]. The primary treatment for HCC involves surgical removal and ablation, followed by targeted kinase inhibitors, anti-angiogenic therapies, immune checkpoint inhibitors, and radiation [2,3]. Despite these treatment options, the 5-year survival rate for liver cancer patients in the United States remains distressingly low, at just 21%, making it one of the most challenging cancer types to manage [4,5]. Several underlying factors can contribute to the development of liver malignancies, including chronic hepatitis virus infections, alcoholic or non-alcoholic fatty liver disease, diabetes, and liver damage caused by exposure to toxic chemicals [6,7,8,9,10,11,12]. Additionally, genetic predisposition plays a role in either promoting or protecting against the development of malignant liver conditions [13,14,15,16,17,18,19,20,21]. For instance, in a model of chemically induced HCC through a single application of diethylnitrosamine (DEN), the speed of malignant transformation varies among different mouse strains [22]. Addressing the pressing need for effective HCC therapies and early diagnosis necessitates a thorough investigation into the factors and mechanisms that predispose individuals to malignant transformation in the liver.

Poly(ADP-ribosyl)ation stands as a pivotal regulatory mechanism governing chromatin remodeling, gene expression, and DNA accessibility [23,24,25,26,27,28]. The synthesis of poly(ADP-ribose) (pADPr) is orchestrated by the poly(ADP-ribose) polymerase (PARP) family of proteins, which in mammals encompasses 17 members, with at least five of them exhibiting poly(ADP-ribosyl)ation activity [23]. Conversely, there is but one enzyme dedicated to the effective degradation of pADPr, known as poly(ADP-ribose) glycohydrolase or PARG [23]. The precise control of pADPr levels is imperative, as disruptions in this pathway can lead to developmental impediments [29,30,31,32]. Moreover, it has been established that PARP proteins are often upregulated, resulting in increased pADPr levels in numerous malignant tumors [33,34,35,36,37]. Decreasing pADPr levels through PARG overexpression has been shown to mitigate the malignant behavior of cancer cells and reduce cancer growth [38,39,40]. PARP1, the most abundant PARP protein in mammalian cells and the principal producer of pADPr, plays a pivotal role in hepatocyte function and has been implicated in the development of liver diseases [41,42,43]. These include conditions like alcoholic or nonalcoholic fatty liver, hepatic injury induced by substances like CCl4 or bile duct ligation, and the initiation of hepatocellular carcinoma (HCC) [44,45,46]. Consequently, the poly(ADP-ribosyl)ating pathway has become a promising area of interest in the study of HCC manifestation.

In this study, we delved into the poly(ADP-ribosyl)ating pathway by focusing on PARG due to its irredundant role. Given the crucial role of pADPr in development, completely knocking out *Parg* leads to early embryonic development issues in mice [32]. To overcome this challenge, we adopted a conditional mouse model approach, resulting in mice with specific *Parg* deletions within hepatocytes. This was achieved by restricting the expression of Cre recombinase to hepatocytes using the albumin promoter and flanking critical *Parg* exons 2 to 4 with recombinase-sensitive loxP sites [47]. While early studies suggested that recombination-induced deletion occurred only in hepatocytes by six weeks of development, subsequent fate-tracing experiments at a single-cell resolution demonstrated efficient recombination soon after birth [48,49]. Consequently, this model allows us to track the final stages of hepatocyte maturation, which culminate during early postnatal development. Our findings demonstrated that PARG depletion from hepatocytes induces morphological and histological abnormalities during early postnatal liver development, marked by extensive hepatocyte apoptosis, acute inflammation, and ductal responses. As development progresses, the liver with hypertrophic hepatocytes transitions to regenerative nodes containing morphologically normal hepatocytes. However, the pronounced anomalies observed in early postnatal development due to PARG deficiency predispose liver tissue to various liver pathologies later in life, including the development of cancer.

## 2. Results

### 2.1. The loxP-Flanked Parg Gene Region Is Deleted in Hepatocytes of Mice Expressing the Albumin Promoter-Driven Cre Recombinase

**Figure 1:**
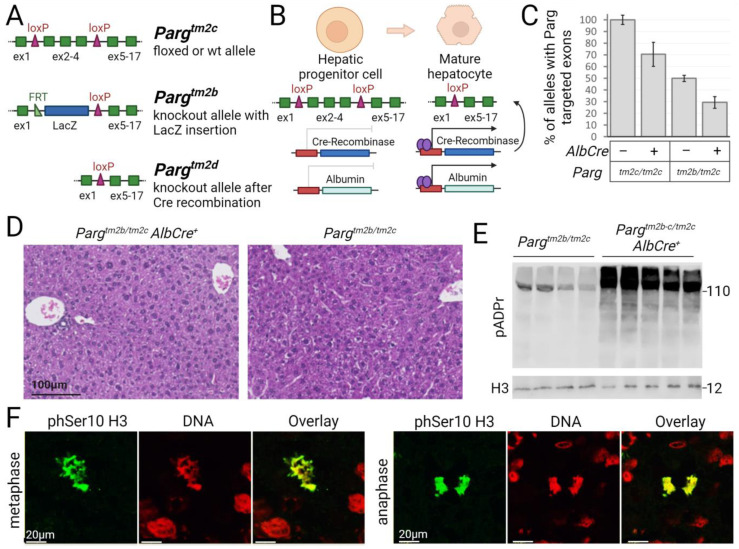
PARG knockout hepatocytes are viable, able to proliferate and accumulates high level of pADPr in vivo. (**A**). Schematic representation of *Parg* alleles and generation of *Parg* conditional knockouts. (**B**). *Parg* critical exons deletion in hepatocytes driven by transgene Cre recombinase expression from albumin promoter. (**C**). Percentage of cells bearing exons 2–4 of *Parg* in liver samples of indicated mice strains. n = 7, data are mean ± SD; *p* < 0.05 between all studied groups. (**D**). Liver histology of adult *Parg* conditional mice. (**E**). Western Blotting for control and *Parg*-knockout livers from 5 weeks old mice stained with antibodies against pADPr showing its increased level in the experimental group. (**F**). Confocal images of *Parg^tm2b/2c^ AlbCre^+^* knockout hepatocytes stained for mitotic mark phSer10H3 (green) and DNA (red) illustrating the ability of *Parg* knockout cells to divide.

The early embryonic lethality observed in *Parg* knockout mice renders this model unsuitable for studying the poly(ADP-ribosyl)ation pathway in liver development and functioning. To address this issue, we utilized a conditional *Parg* knockout system. Specifically, we crossed mice in which crucial *Parg* exons 2 to 4 were flanked by loxP sites with mice expressing Cre recombinase under the control of the albumin promoter. This genetic approach facilitated Cre-driven deletion of *Parg* exons specifically in hepatocytes [47,50] (Figure 1A,B).

Our initial objective was to determine whether *Parg*-targeted exons were deleted in liver cells with Cre recombinase expression controlled by the albumin promoter (Figure 1A,B). We employed standard PCR with primers flanking the loxP sites and observed the expected 850bp band for deleted *Parg* exons, validating recombinase activity at the intended site (Supplemental Figure S1). To assess recombination efficiency, we conducted qPCR using primers within the deleted region and a reference region. This method enabled us to quantify the deleted *Parg* alleles in *Parg^tm2c/tm2c^* mutants or the complete knockout of *Parg* in the livers of *Parg^tm2b/tm2c^* mutants. Initially, we confirmed the functionality of our method in heterozygous *Parg^tm2b/tm2c^* mice, where the loxP-flanked PARG region is replaced by a LacZ cassette. The qPCR results indicated a twofold reduction in the targeted region compared to wild type or *Parg^tm2c/tm2c^* mice (Figure 1C and Supplemental Figure S2). Subsequently, we analyzed experimental *Parg^tm2c/tm2c^* and *Parg^tm2b/tm2c^ AlbCre^+^* mice and observed *Parg* knockout in 30–50% of alleles or cells (Figure 1C). We repeated the experiment with additional samples. Histological analysis of livers for 15-week-old animals did not reveal significant changes (Figure 1D). We expected that the deletion of the PARG protein in hepatocytes would lead to an increase in pADPr levels. Western Blotting analysis revealed a significant increase in pADPr levels in the livers of *Parg* knockout 5–6 weeks old mice, confirming the efficient deletion of the PARG protein (Figure 1E, Supplemental Figure S3). To determine whether hepatocytes without PARG could divide mitotically, we stained liver sections with antibodies against the mitotic chromatin marker phSer10H3 histone modification (Figure 1F). We observed the presence of dividing *Parg* knockout hepatocytes in the liver tissue.

**Figure 2:**
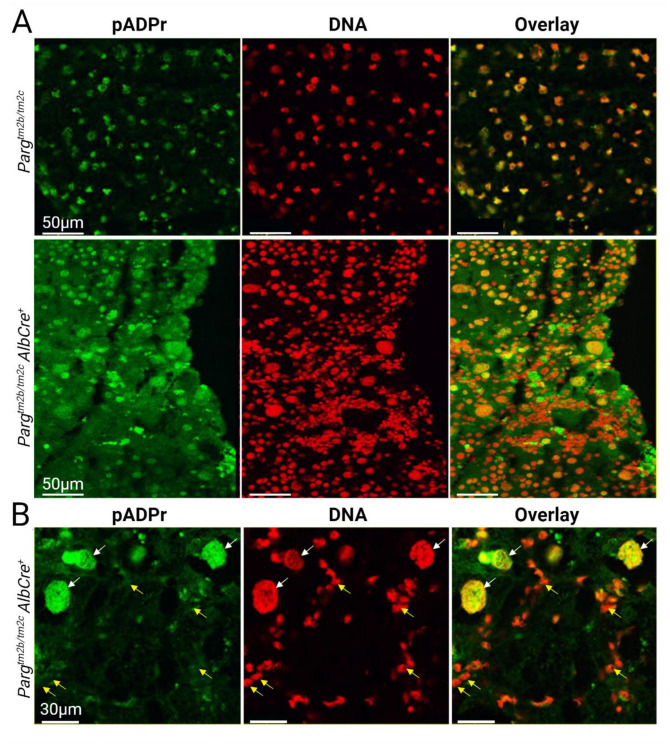
pADPr is high in hepatocytes of *Parg* knockout and control animals and low in mononuclear cells. (**A**). Confocal images of control and *Parg* knockout liver stained for pADPr with special reagent (green) and DNA with Draq 5 (red). pADPr is seen in nuclei of hepatocytes in both groups. (**B**). Confocal images of *Parg^tm2b/2c^ AlbCre^+^* knockout hepatocytes stained for pADPr (green) and DNA (red). The level in hepatocytes nuclei (white arrows) is higher than in mononuclear cells nuclei (yellow arrows).

To investigate changes in pADPr at the cellular level between control and *Parg*-liver knockout mice (Figure 2), we performed immunofluorescence staining of liver sections using antibodies. Initially, pADPr did not stain with either anti-pADPr antibodies or a special reagent when tissues were pre-fixed. However, when we stained unfixed flash-frozen tissue, pADPr was detected in the nuclei of both control and experimental livers. Overall, we did not find changes in the level of pADPr in individual hepatocyte nuclei between the two groups. Additionally, we demonstrated that pADPr is more abundant in hepatocyte nuclei compared to nuclei of cells in the surrounding stroma (Figure 2B), making them the primary contributors to pADPr levels in bulk samples.

### 2.2. The Regeneration Capacity of Parg Hepatocyte-Knockout Livers Is Not Compromised

The liver possesses a remarkable ability to regenerate itself following the loss of hepatocytes, primarily through their proliferation. We aimed to investigate whether the knockout of *Parg* affects the hepatocytes’ proliferation capability and the liver’s ability to regenerate. Liver damage was induced by intraperitoneal injection of CCl4, and liver histology was examined at 2- and 7-days post-injection. We observed that both control and *Parg* knockout livers exhibited similar liver damage and hepatocyte necrosis in the periportal area two days after injection. However, at 7 days post-injection, both the control and experimental groups successfully regenerated liver mass and restored normal liver morphology (Supplemental Figure S4). This indicates that the absence of PARG does not impair the hepatocytes’ ability to proliferate.

### 2.3. Liver Development and Functioning in the Absence of PARG in Hepatocytes Is Affected

**Figure 3:**
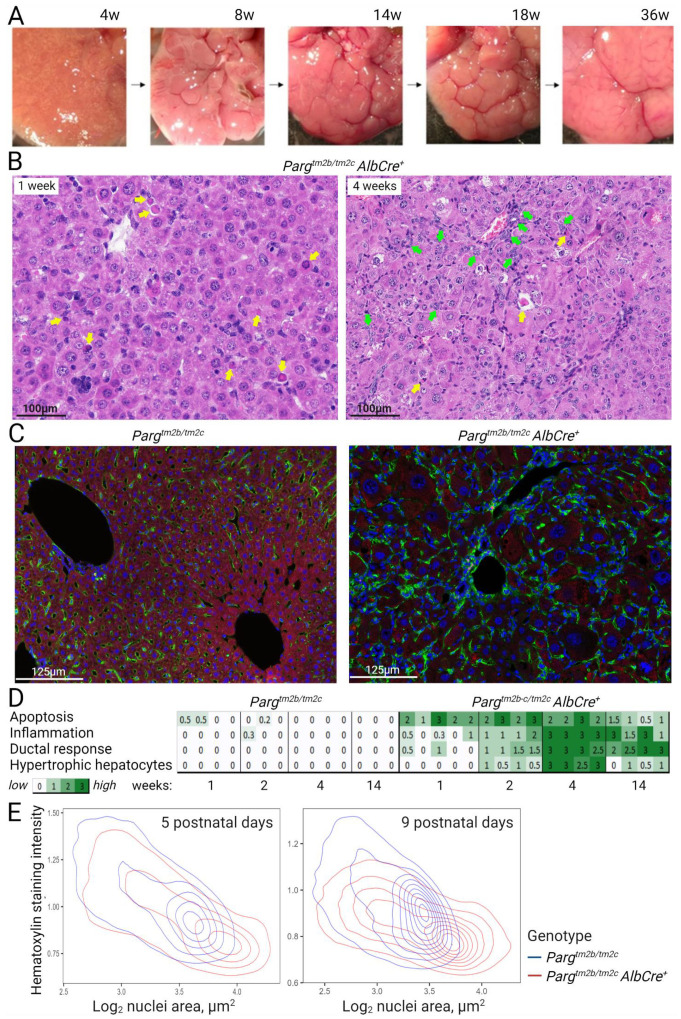
The *Parg* knockout affects liver maturation in mice. (**A**). Liver morphology at stated postnatal weeks of age for *Parg^tm2bc/tm2c^ AlbCre^+^* mice, the left lobe is shown. The nodes become noticeable at 4 weeks of postnatal development, continue to grow at 8–14 weeks and eventually replace the total liver mass. (**B**). Extensive hepatocyte apoptosis in the centrilobular (central vein, CV) region and an intense ductal response with inflammation in periportal (PT) region were detected around first and forth weeks of postnatal development correspondingly in *Parg^tm2b/2c^ AlbCre^+^* mice. H and E staining of livers are shown at 200× magnification. Apoptotic cells are marked with yellow arrows, ductal response with green arrows. (**C**). Confocal images of control and *Parg* knockout liver autofluorescence (red) and immunofluorescence staining of Sinusoids with anti-mouse antibodies for endogenous mouse antibodies (green), and DNA with Draq 5 (blue) at the same magnification. Enlargement of hepatocytes and their nuclei is obvious in the livers of experimental mice. (**D**). Histopathological scoring of liver tissue based on distinct characteristics, ranked from from 0 (not observed) to 3 (severe), in control and *Parg* knockout mice at specified weeks after birth. (**E**). Nuclei areas were selected on H and E stained sections from control and PARG knockout livers on days 5 and 9 of postnatal development using the StarDist neuronal network. Hematoxylin intensity and single nuclei areas were plotted. The increase in size and decrease in hematoxylin staining were noticed for the experimental group at both developmental time points.

We initiated our investigation by conducting a histological assessment of *Parg* knockout livers. The liver morphology appeared similar between control and knockout mice during the first two weeks of postnatal development. Over the subsequent four weeks, *Parg* knockout livers began to pale in comparison to control livers. Spherical nodules emerged within the liver tissue, started to grow, and by 9–11 weeks of postnatal development, they had completely replaced the original liver tissue (Figure 3A). Upon histological analysis, it became evident that at 1 week of postnatal development, there was an elevated level of hepatocyte apoptosis, primarily in centrilobular areas, within the livers of *Parg* knockout mice. Concurrently, an intense ductal response in periportal areas and inflammation became noticeable, beginning at this time and accelerating by the fourth week when the liver tissue became hypertrophic, with significantly enlarged hepatocytes (Figure 3B–D).

**Figure 4:**
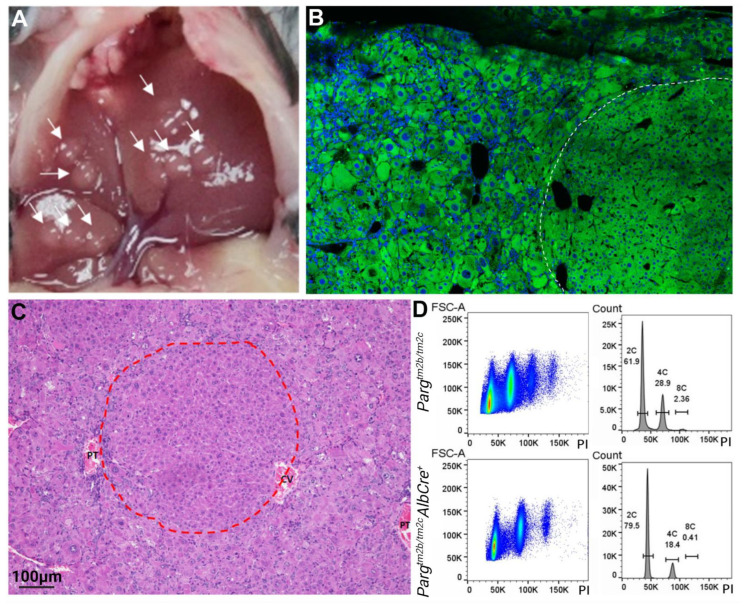
The nodes in the liver of *Parg* conditional mice with two AlbCre allele represent the areas of healthy hepatocytes growing clonally. (**A**). Mutating *Parg* induces tissue nodes (white arrows) formation in mutant livers. (**B**). Confocal images of *Parg^tm2ctm/2c^ AlbCre^+^* (5 weeks old) liver tissue: cytoplasm (green) and DNA stained with Draq 5 (blue). The node is circled in white. (**C**). H and E staining. The node is circled in red. Portal triad PT, central vein CV. (**D**). Nuclei were isolated from livers of control and *Parg* knockout mice 8 weeks old, stained with PI for DNA, and flow cytometry analysis was performed. Representative samples are shown. More polyploid nuclei are present in control livers. Moreover, nuclei from the experimental group demonstrate higher uptake of DNA dye PI, which is recognized by higher fluorescence intensity.

However, when we analyzed early postnatal stages, specifically up to two weeks of development (postnatal days 5 and 9), by selecting single nuclei and calculating their areas, we detected an increase in nuclei size in *Parg* knockout mice across all the studied stages (Figure 3E). Additionally, histological analysis revealed that the spherical nodules, which were initially detected during the morphological examination of the liver and continued to grow with age, represented normal liver tissue with healthy-looking hepatocytes. These nodes seemed to possess a clonal nature and displayed advantageous traits that enabled them to overcome the deficiency of PARG (Figure 4A–C). It is plausible that these cells could circumvent *Parg* recombination and restore the protein level. However, when we performed qPCR analysis to check whether *Parg* exons were excised in these “survived” tissues, it indicated efficient recombination (Supplemental Figure S2C). It is possible that other mutations or epigenetic changes provide these hepatocytes with advantages for growth and proliferation.

To assess the ploidy of nuclei in developing liver tissue, we isolated single nuclei, stained them with the DNA marker PI, and conducted flow cytometry analysis. While we did not detect an increase in ploidy in *Parg* knockout livers, we did observe an increase in DNA staining with PI dye in the nuclei of the experimental group (Figure 4D). This finding could suggest a more open chromatin state in these cells.

### 2.4. The Lack of PARG in Hepatocytes Predisposes Liver to Tumors

Poly(ADP-ribosyl)ating pathway is known to play an essential role in cancer development. To investigate the tumor-forming potential of liver cells lacking PARG, we employed a chemically-induced liver cancer model. Mice were injected with diethylnitrosamine (DEN) 14–16 days after birth and were monitored for cancer development. The early mouse death during first weeks after DEN injection was similar between groups: 27.6% for control and 31.8% for *Parg* knockout group.

**Figure 5:**
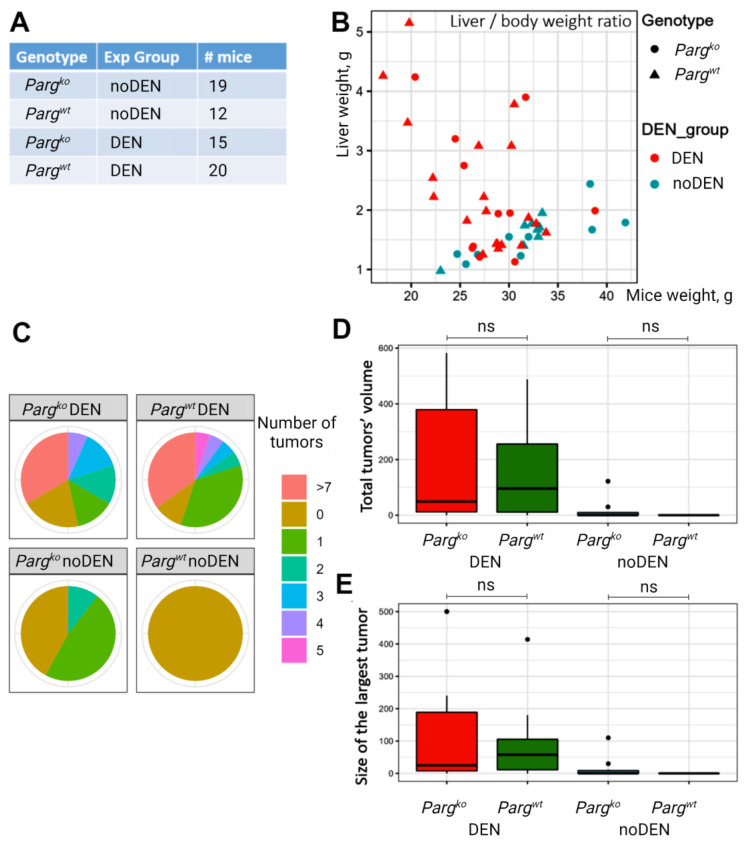
*Parg* knockout in hepatocytes promotes liver tumorigenesis. (**A**). Number of mice used per experimental group. (**B**). Liver and body weight plotted for control and *Parg* hepatocyte-knockout mice with/without DEN injected. (**C**–**E**). The pie charts for the number of tumors (**C**), total tumor volume (**D**), and size of maximum tumor (**E**) per liver in studied groups. No statistical significance was found between *Parg^wt^* and *Parg^ko^* mice within the DEN and noDEN groups using the ANOVA single-factor test.

**Figure 6:**
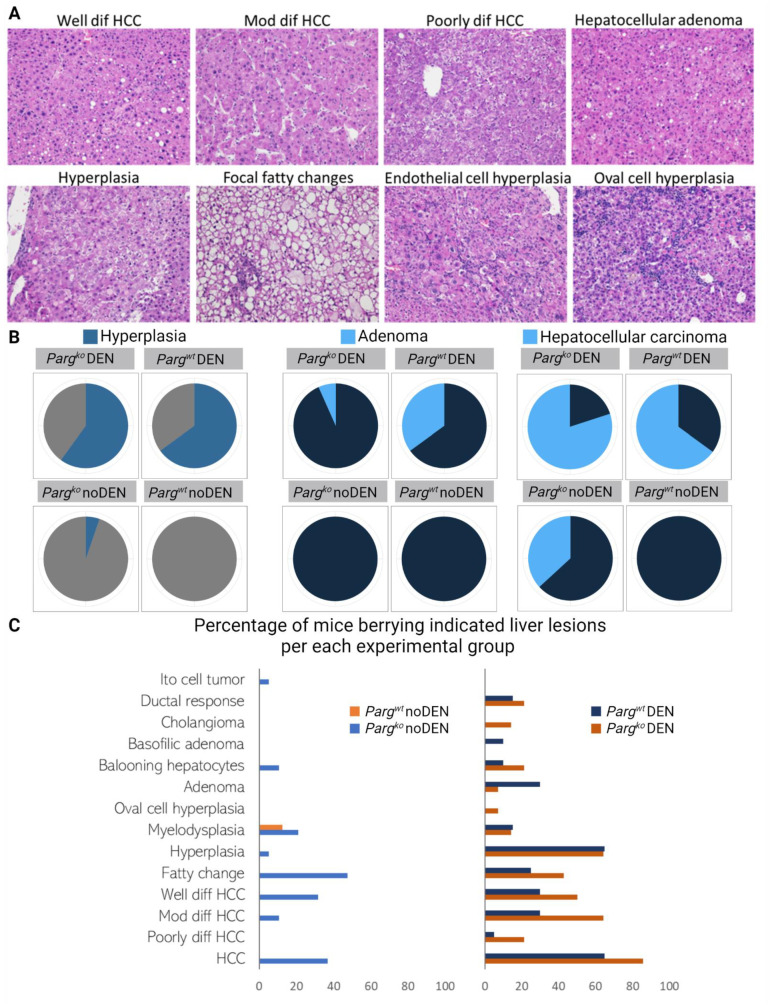
The knockout of *Parg* exacerbates DEN-induced carcinogenesis and enhances the development of malignant conditions in intact animals. (**A**). Histological analysis of livers from mice induced with DEN revealed different pathologies. H and E staining of lives sections, representative images with 200× magnification. (**B**). Liver lesion types were identified on histological slides. The number of mice carrying liver hyperplasia, adenoma, and hepatocellular carcinoma was counted for each experimental group and plotted as a pie chart. (**C**). Percentage of animals bearing different types of liver malformations per total number of animals in a study group.

Both control and *Parg* knockout mice originated hyperproliferative nodules. While the liver-to-body weight ratio significantly increased in DEN-injected mice, there was no significant difference observed between the control and *Parg* knockout experimental groups (Figure 5A,B). Although the number of nodules or the size of the largest tumor did not exhibit significant differences between these two groups, there was a tendency for the *Parg* knockout livers to display a higher occurrence (Figure 5C–E). Samples from all nodules were collected, and a thorough histological evaluation was performed. The analysis revealed a wide variety of liver proliferative lesions in the DEN-injected groups, with a notable prevalence of hyperplasia, adenoma, and hepatocellular carcinoma (Figure 6A,B). Hyperplasia and adenoma were more frequently observed in the DEN-treated control group, whereas hepatocellular carcinoma tumors were more predominant in the DEN-treated *Parg* knockout group (Figure 6B,C). Moreover, cases of hepatocellular carcinoma and other liver histological abnormalities, such as fatty changes, hyperplasia, myelodysplasia, and cholangioma, were detected in *Parg* knockout mice even in the absence of DEN treatment. The wide variety of lesions belonging to the hepatocyte lineage suggests an alteration at an early stage of hepatocyte development.

## 3. Discussion

In our current study aimed at investigating the regulation of hepatocytes by the poly(ADP-ribosyl)ating pathway, we explore the impact of PARG, an enzyme responsible for degrading pADPr, on hepatocyte maturation during postnatal liver development and its potential role in hepatobiliary carcinogenesis. In our *Parg* conditional knockout mouse model, we employ mice expressing Cre recombinase under the control of the albumin promoter [47]. While early reports suggested that the recombination peak occurs by week 6 of postnatal development and is insufficient in earlier stages, later reports revealed the effectiveness of Cre recombination starting embryonically [48,49]. The albumin promoter exhibits high expression in hepatocytes during their differentiation from progenitor cells into cholangiocytes and hepatocytes, followed by its silencing in cholangiocytes and significant activation in hepatocytes [47,48,51]. This critical transition occurs in the late stages of embryonic and early postnatal development [48,51]. Concurrently, hepatocytes undergo final maturation, silencing fetal genes, and activating mature hepatocyte genes responsible for essential liver functions such as P450 enzymes and other metabolic processes, particularly those involved in bile acid, retinol, and xenobiotic metabolism [52,53,54].

Our findings demonstrate that PARG plays a crucial role in the later stages of hepatocyte maturation. We observe significant hepatocyte loss by apoptosis during the first week of postnatal development in *Parg* knockout livers. The liver possesses remarkable regenerative capabilities, tightly regulating hepatocyte numbers [55]. When extensive hepatocyte loss occurs, the remaining hepatocytes actively proliferate to restore liver volume [56]. However, in the case of intensive hepatocyte apoptosis in *Parg* knockout livers, an alternative strategy for restoring liver function initiates. The remaining hepatocytes begin to enlarge, a noticeable change by week one of postnatal development, leading to the formation of hypertrophic tissue with some enlarged hepatocytes by weeks four to six [57,58]. This period also sees the emergence of an inflammatory response and the initiation of an acute ductal response, a process involving the active proliferation of ductal cells: hepatic progenitor cells, or cholagioblasts. Starting at three weeks of postnatal development, we observe the emergence of regeneration nodes in the liver of *Parg* hepatocyte-knockout mice, characterized by morphologically normal hepatocytes and liver tissue structure, devoid of ductal response-associated inflammation. These hepatocytes do not display the hypertrophic phenotype, instead, they divide and eventually replace the entire liver tissue. This type of clonal regenerative nodes rescuing liver function also occurs in several other knockout genetic models [52,59]. It was shown that this surviving tissue represents the clonal expansion of mutated hepatocytes that managed to evade recombination and restore the initial levels of the targeted protein, however, other compensatory mechanism could also be involved [52,59]. We demonstrate that PARG-targeted recombination happened in these nodes. It is possible that they originate from hepatocytes that were finished the maturation process before the PARG depletion and are able to proliferate. We could also suggest that additional genomic or epigenetic alterations could happen, probably affecting pADPr levels. Thus, while we observe an increase in pADPr levels in the livers of 5-week-old mice, pADPr levels return to those of control animals by 11 weeks or in older animals.

It is noteworthy that this type of regeneration, involving ductal response and differentiation of cells from progenitors or cholangioblasts to hepatocytes, is not commonly observed in all other scenarios of hepatocyte loss, such as partial hepatectomy, CCl4 or DDC diet-induced injury, but has been demonstrated in cases of choline-deficient-ethionine-supplemented diet, specific genetic perturbations, in cholestatic injury, and senescent hepatocytes-induced injury [60,61,62,63,64]. Ductal reactions play an increasingly vital role in understanding hepatic stem and progenitor cells during liver regeneration, the processes driving hepatic fibrosis, and the development of hepatobiliary carcinogenesis [65]. In humans, ductal responses have been identified in several liver disorders, including cholangitis, alcoholic and non-alcoholic hepatitis and steatohepatitis, hepatitis B and C, all of which are known to promote the initiation and development of hepatocellular carcinoma (HCC) [65]. *Parg* liver knockout mice could serve as a valuable model for studying ductal responses as they are viable, fertile, easy to breed, and reliably develop ductal responses early in life, saving time and resources in studying this process.

The cause of such dramatic changes in early postnatal liver development and hepatocyte differentiation during these stages can be attributed to the poly(ADP-ribosyl)ating pathway’s role in chromatin remodeling, thereby regulating gene expression [23,25,26]. Using the model organism *Drosophila melanogaster*, we have shown that PARP1 localizes to gene promoters, influencing their activity by activating developmental genes and dampening the expression of metabolic genes [66,67]. PARG has seemingly opposite role to PARP1, however, they mainly work in cooperation. PARG is essential for maintaining PARP1 activity by removing pADPr from it. In the absence of PARG, PARP1 becomes auto-modified and relocates to Cajal bodies. Consequently, when we remove PARG from the poly(ADP-ribosyl)ating pathway, we also deactivate PARP1 [68].

The poly(ADP-ribosyl)ating pathway plays a significant role in cancer development. While most research has focused on the role of PARP1 in malignant transformations, fewer investigations have delved into the role of PARG. Interestingly, in hepatocellular carcinoma, high levels of both PARP1 and PARG are associated with an unfavorable prognosis. Notably, the expression of both proteins positively correlates with cancer grade, potentially influencing pADPr turnover rates rather than levels.

To examine the effect of PARG depletion on liver carcinogenesis, we induced HCC chemically by a single DEN injection in 2-week-old mice. *Parg* knockout livers exhibit various liver abnormalities and develop random malignancies later in life, even in the uninduced group, and have a tendency to worsen cancer incidence and severity. It is plausible that the pathological events observed in PARG-depleted livers during early postnatal development predispose them to cancer development.

Around 15–20% of all cancer cases are preceded by infection, chronic inflammation or autoimmunity at the same tissue or organ site [69,70]. Tumors initiate in two steps: the accumulation of mutations or epigenetic changes in genes related to oncogenic pathways, and the creation of malignant clones. The early development alterations in *Parg* mutant livers contributed to the occurrence of both events. Inflammation produces reactive oxygen and nitrogen species by inflammatory cells, promotes mutagenesis and activates epigenetic machinery in cells [71,72,73]. The intensive hepatocyte loss and clonogenic nature of early repair favor the appearance and amplification of mutated hepatocytes. In addition, inflammatory signaling could contribute to the outgrowth of transformed clones into frank tumors by promoting pro-survival pathways and creating a microenvironment favorable for tumor growth [74,75].

Previous studies have shown that PARG depletion in adult mice livers, induced by virus-introduced Cre recombinase, reduces HCC development induced by a single DEN injection followed by serial CCl4 injections [76]. These results are not contradictory to the findings in our current study. In our model, PARG depletion occurs around birth and affects the final stages of hepatocyte maturation, whereas the study by Yu et al. involved PARG depletion from fully differentiated hepatocytes. Moreover, their work suggests that PARG is not essential for fully differentiated hepatocytes, supporting the results obtained in our study as PARG depletion from rescued hepatocytes does not affect their normal functioning and proliferation after chemically induced liver repair.

Furthermore, our research demonstrates that PARG-depleted hepatocytes accumulate more dye in the nucleus when stained with markers such as propidium iodide, suggesting that chromatin remains in a more open state. Our previous work has shown that PARG may play a role in gene silencing. Chip-seq analysis in Drosophila melanogaster revealed PARG enrichment in gene bodies for several genes, with its presence required for their silencing during development [77,78]. It is plausible that removing PARG leads to a more open chromatin state, allowing better access for DNA dyes.

In conclusion, our study sheds light on the critical role of the poly(ADP-ribosyl)ating pathway in the last stages of hepatocyte maturations. Affecting this process by PARG depletion results in liver pathologies early in life a predispose it for malignancies in future. Understanding these processes is essential for advancing our knowledge of liver biology and addressing liver-related diseases, including cancer.

## 4. Materials and Methods

### 4.1. Mice

The mouse strain used for this research project, C57BL/6N-Atm1Brd Pargtm2a(KOMP)Mbp/JMmucd, RRID:MMRRC_048977-UCD, was obtained from the Mutant Mouse Resource and Research Center (MMRRC) at University of California at Davis, an NIH-funded strain repository, and was donated to the MMRRC by The KOMP Repository, University of California, Davis; Originating from Stephen Murray, The Jackson Laboratory. The mouse strain used for this research project, C57BL/6N-Tg(CAG-Flpo)1Afst/Mmucd, RRID:MMRRC_036512-UCD, was obtained from the Mutant Mouse Regional Resource Center, a NIH funded strain repository, and was donated by the MMRRC at UC Davis. The original transgenic was donated by Dr. Konstantinos Anastassiadis from Technische Universitaet Dresden. The PARG conditional mice PARG^tm2c/tm2c^ were generated by crossing Pargtm2a(KOMP)Mbp/JMmucd and C57BL/6N-Tg(CAG-Flpo)1Afst/Mmucd to induced the FLP recombination of LacZ cassette. The final *Parg^tm2c^* allele has exons 2–4 flanked with loxP sites and *Parg^tm2b^* allele has exons 2–4 replaced by LacZ cassette. AlbCre mice (Jax Strain 003574) that express Cre recombinase under the albumin promoter were crossed with PARG^tm2b/tm2c^ or PARG^tm2c/tm2c^ mice for the deletion of PARG critical exons 2–4 in hepatocytes. Mice were genotyped using regular PCR for *Parg^tm2c^* allele with CSD-Parg-F1 TGGGTCCTTCAGGATGCTTTCTGTGATA and CSD-Parg-ttR1 GGGCTGGAGAGATGTCTCAGTGGTTAAA primers, for AlbCre transgene presence with 20239 TGCAAACATCACATGCACAC, 20240 TTGGCCCCTTACCATAACTG and oIMR5374 GAAGCAGAAGCTTAGGAAGATGG primers. *Parg^tm2b^* mice were genotyped with TaqMan qPCR as described previously with LacZ_F1 CTACGGGTAACAGTTTCTTTATGG, LacZ_R1 CGTTCAGACGTAGTGTGACG primers and LacZ_P1 TGAAACGCAGGTCGCCAGCGG Hex labeled probe [79]. To detect the cut of targeted PARG exons regular PCR was used with the following CSD-Parg-F1 TGGGTCCTTCAGGATGCTTTCTGTGATA, CSD-Parg-3A CCAGGGCAGGGCGAGCCATAAATAC primers, and qPCR with PARG_ex234_F2 ACAGGAGGAGGTGGATGTG, PARG_ex234_R2 GCTGCTTTCTTGTCCAGTCA primers, and PARG_ex234_P2 TCCAGTTCCAATGTCCTCGGCAC Fam labeled probe inside the cut region along with Ref_F1 ACGGAGATCGTTGCCATTG, Ref_R1 GCTGCCAGATGAACTCGGA primers, and Ref_P1 ACGCACTTCACTTCAGACGCTACCT Hex labeled probe as a reference [79]. All animal experiments were approved by the University of North Dakota Institutional Animal Care and Use Committee (IACUC).

### 4.2. Western Blotting

Western Blotting was performed as described previously [38]. Briefly, RIPA buffer (Thermo Fisher, Waltham, MA, USA) was added to tissue samples in 1:15 ratio and homogenized with glass pestle. After 10 min on ice, it was centrifugated for 5 min at 14,000 rpm, then 350 µL of supernatant mixed with 350 µL of 2× sample loading buffer SLB 2× (Biorad, Hercules, CA, USA) and heated at 99 °C for 5 min. Proteins were separated in 14% PAAG gel and transferred to nitrocellulose membrane. Antibodies used were against pADPr (sc-56198, Santa Cruz, Dallas, TX, USA), H3 (sc-10809, Santa Cruz, Dallas, TX, USA), anti-mouse HRP (G21040, Invitrogen, Waltham, MA, USA), anti-rabbit HRP (Perkin Elmer, Waltham, MA, USA).

### 4.3. Chemically Induced Liver Tumor Generation

To induce liver cancer, mice at 14–16 days after birth were intraperitoneally injected with 20 mg/kg diethylnitrosamine (Tokyo Chemical Industry, Tokyo, Japan) (experimental, DEN group) or DMSO/PBS (control, noDEN group). After tumor development, livers were assessed morphologically and weighed. Normal tissue and morphologically abnormal lesions were submitted for histological analysis. Experienced histopathologists analyzed samples and identified the types of pathologies.

### 4.4. Tissue Histology and Immunofluorescence Staining

Preparation of histological slides preparation was performed by the UND histological core. For developmental studies, 4–6 mice per developmental time point were analyzed. Liver samples were fixed in 10% formalin for 8 h, embedded in paraffin, and cut into 8–12 um sections. Slides with sections were stained with hematoxylin and eosin, mounted with Permount mounting medium, and imaged with a Hammamatsu NanoZoomer 2.0HT Digital Slide Scanner. For immunofluorescence staining, liver samples were fixed in 4% PFA for 30 min and submerged in 20% sucrose (Sigma Aldrich, St. Louis, Missouri, MO, USA), or directly flash frozen in OCT Compound (Thermo Fisher) for cryosectioning into 12 um slices. Sections on slides were stained with a special reagent against pADPr (MABE1031, Sigma Aldrich) or antibodies against phSer10H3 (#9701, Cell Signaling), a marker of mitotic chromosomes, secondary antibodies anti-mouse Alexa 488 (A28175, Invitrogen), anti-rabbit Alexa 568 (A-11011, Invitrogen), and DNA marker Draq 5 (62251, Thermo Fisher). The pathology assessment of the liver tissue was performed by an experienced histopathologist while closely following the nomenclature found in the National Institute of Environmental Health Sciences: https://www.niehs.nih.gov/research/resources/visual-guides/liverpath/index.cfm accessed on 12 September 2022.

### 4.5. Nuclei Area Calculation in Liver Sections

To calculate the nucleus or cell areas on histological hematoxylin and eosin sections, QuPath 0.4.0 software was used with deep-learning neural network StarDist. Staining was separated into hematoxylin and eosin channels, and nuclei were identified in the hematoxylin channel using a StarDist with model, pre-trained on H and E sections.

### 4.6. Nuclei Isolation from Liver and Flow Cytometry

Nuclei were isolated as described previously. Briefly, liver tissue was homogenized in nuclear extraction buffer and spun through a mesh filter. Nuclei pellets were washed in a washing buffer, fixed with ice-cold 70% ethanol for 30 min, and washed in PBS with 0.5% BSA. After staining DNA with FxCycle PI kit (F10797, Thermo Fisher), nuclei were subjected to flow analysis with BD FACS Symphony A3.

### 4.7. Statistical Analysis

Statistical analysis was conducted with a two-sided Student’s *t*-test or ANOVA test, and *p*-value < 0.05 was considered to be statistically significant.

## Data Availability

Data generated or analyzed during this study is available upon request.

## Supplementary Materials

The following supporting information can be downloaded at: https://www.mdpi.com/article/10.3390/ijms242417205/s1.
