# Supplementary material for: Disrupting Poly(ADP-ribosyl)ating Pathway Creates Premalignant Conditions in Mammalian Liver
Source: Int J Mol Sci. 2023 Dec 6;24(24):17205. doi: 10.3390/ijms242417205 (PMC10743425; doi:10.3390/ijms242417205)
Supplement: Supplementary file 1 [file ijms-24-17205-s001.zip › ijms-2718357-supplementary.pdf]

International Journal of
Molecular Sciences

MDPI

Article

Disrupting poly(ADP-ribosyl)ating pathway creates premalignant conditions in mammalian liver

Yaroslava Karpova [1,2], David J. Orlicky [3], Edward E. Schmidt [4] and Alexei V. Tulin [1]*.

1 Department of Biomedical Sciences, School of Medicine and Health Sciences, University of North Dakota, 501 North Columbia Road, Grand Forks, ND 58202, USA; iaroslava.karpova@und.edu
2 Koltzov Institute of Developmental Biology of Russian Academy of Sciences, Moscow 119334, Russia
3 Department of Pathology, University of Colorado School of Medicine, Aurora, CO, USA; david.orlicky@cuanschutz.edu
4 Microbiology & Cell Biology, Montana State University, Department of Microbiology & Immunology, Lewis Hall, Bozeman, MT 59718, USA; eschmidt@montana.edu
5 Redox Biology Laboratory, University of Veterinary Medicine, Budapest 1078, Hungary; edward.schmidt@univet.hu
* Correspondence: alexei.tulin@und.edu

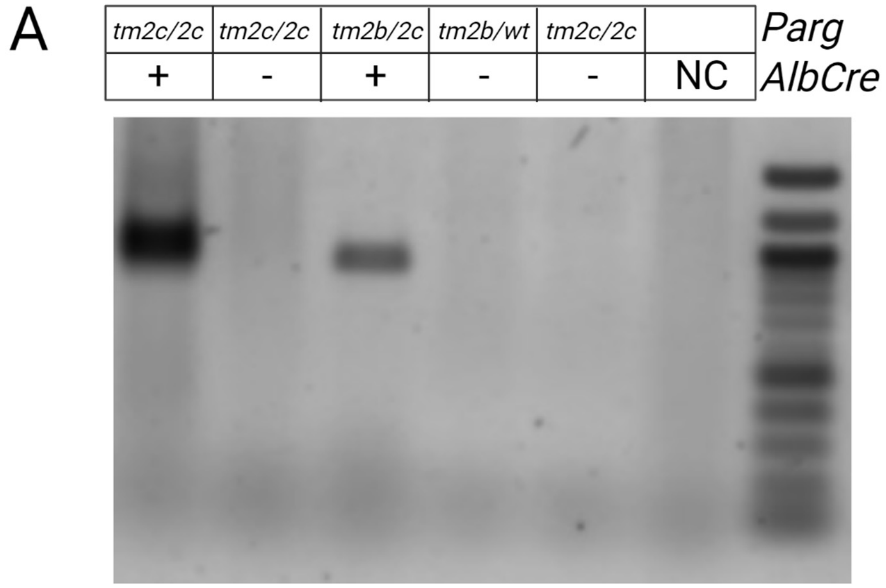

Supplemental Figure S1. PCR to detect the *Parg* targeted exons deletion in *PARG*$^{tm2b\text{-}c/tm2c}$ *AlbCre*$^{+}$ mice. Amplification with primers flanking the targeted region demonstrated the presence of band of correct size.

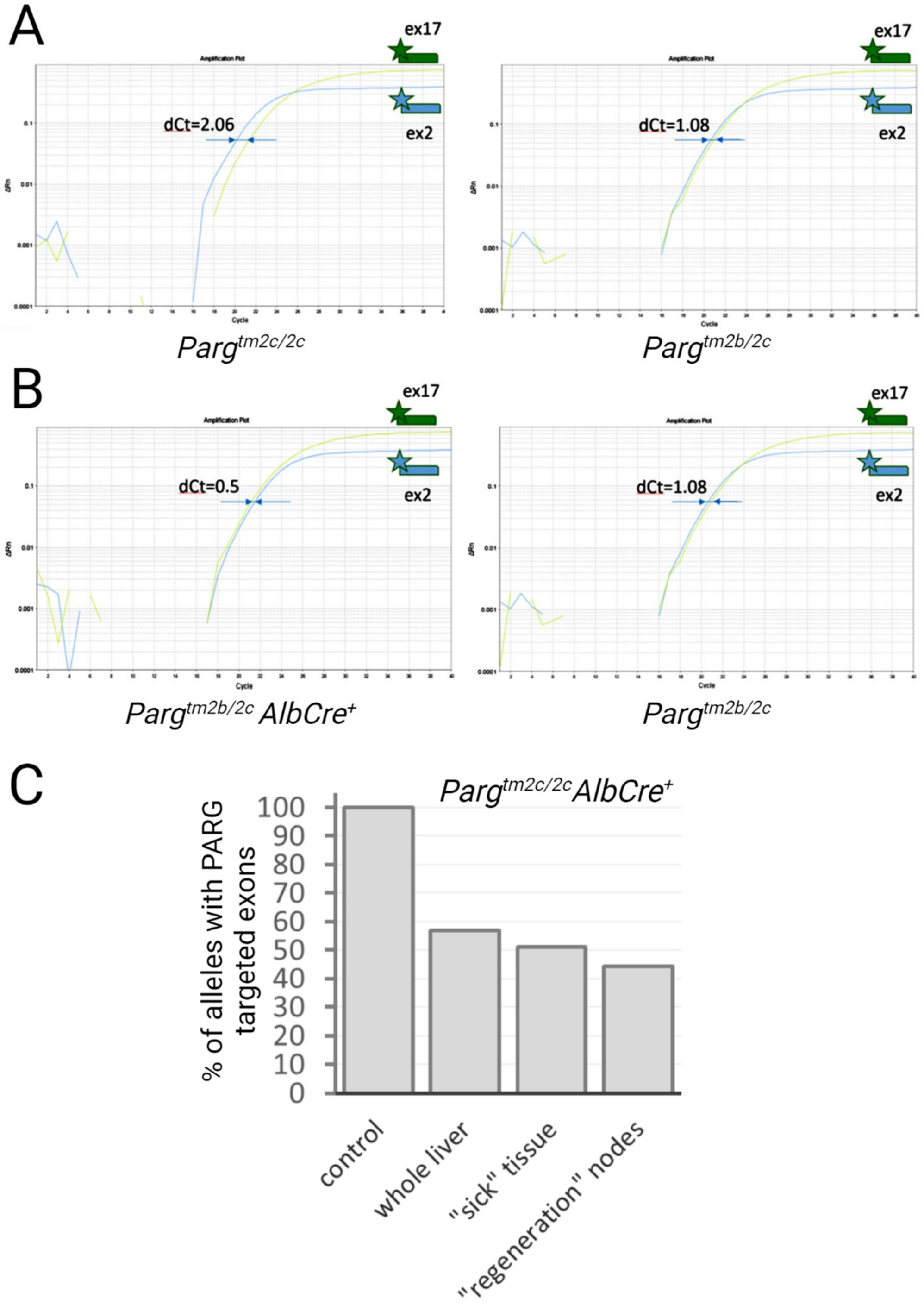

Supplemental Figure S2. *Parg* exons 2 to 4 are deleted in liver of experimental *Parg*$^{tm2c/tm2c}$*AlbCre*$^{+}$ mice. Taqman qPCR-based assay to detect the *Parg* targeted region deletion in experimental mice. Two sets of primers with probes were used, one in the targeted region, and the other in the reference undisturbed region. The difference between amplification dCt will indicate the relative amount of cut DNA. **A.** *Parg*^{tm2b} knockout allele where PARG targeted exons are replaced by Lac0Z cassette, the decrease in Ct is recorded. **B.** Cre recombinase expressing *Parg* conditional mice demonstrate the decreased amplification of *Parg*-targeted region indicating the recombinase-induced deletion. **C**. Percentage of cells bearing *Parg* targeted exons in whole liver, “sick” tissue with hypertrophic hepatocytes or “regeneration” nodes samples in *PARG*$^{tm2c/tm2c}$ *AlbCre*$^{+}$ mice.

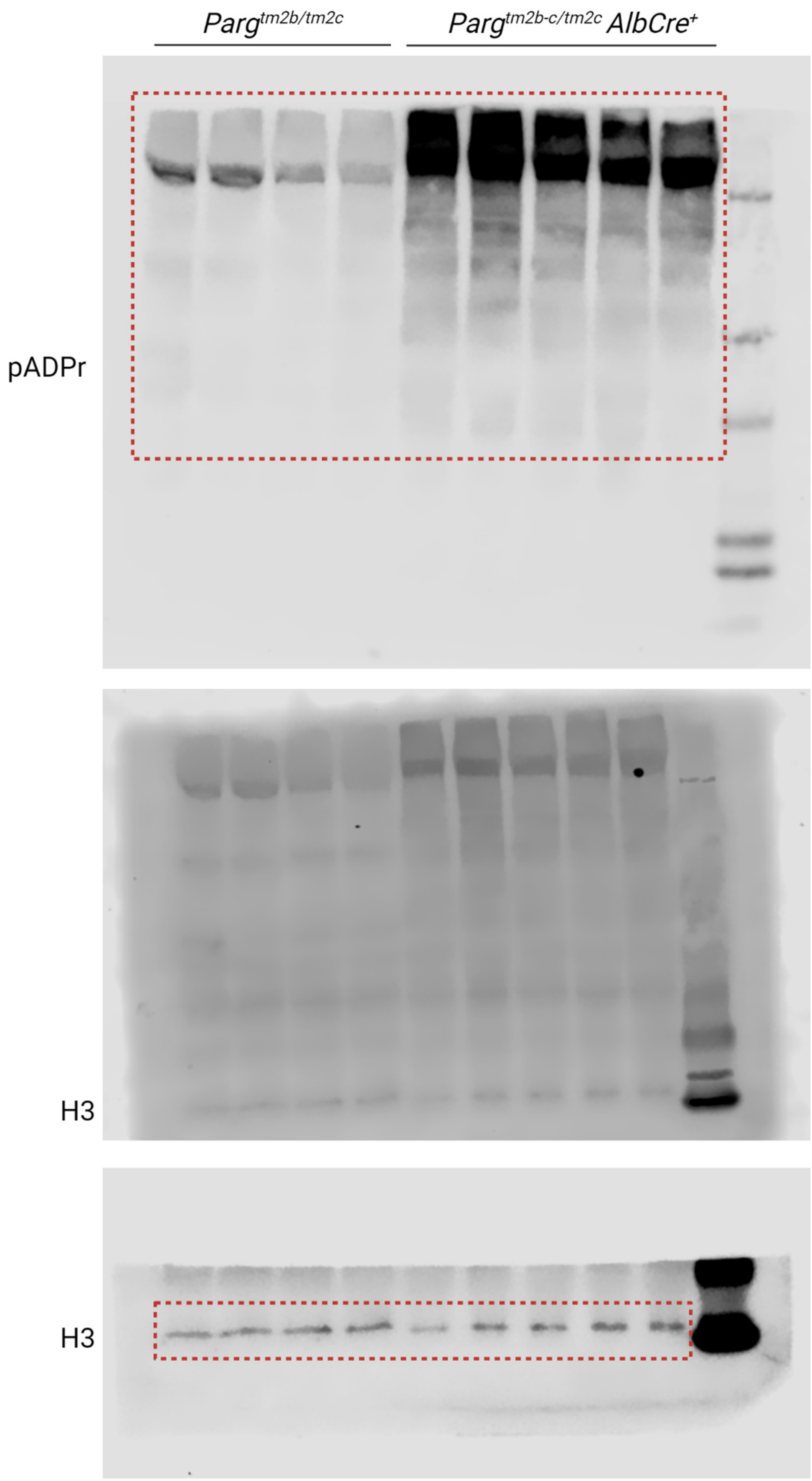

Supplemental Figure S3. Uncropped versions of Western Blots from Fig. 1E. For bottom panel top part of membrane was covered for increased visibility. Whole blot is present on middle panel.

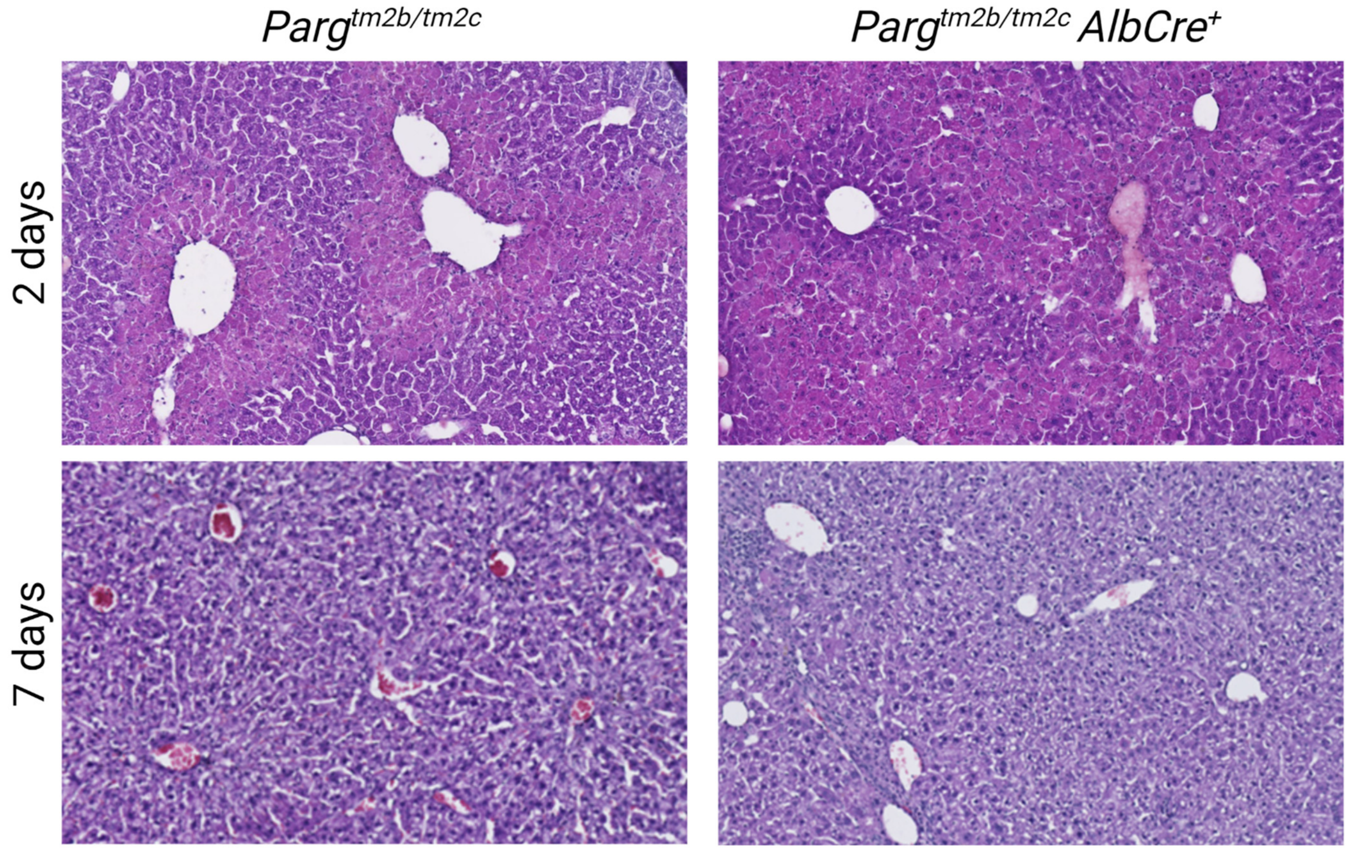

Supplemental Figure S4. Histological analysis of regeneration abilities control *Parg*$^{tm2b/tm2c}$ and mutant *Parg*$^{tm2c/tm2c}$ *AlbCre*$^{+}$ mice liver two and seven days after CCl4 chemical induction. Similar level of liver damage and regeneration process was demonstrated for two studied mice strains.
